# Systemic treatment in patients with malignant pleural mesothelioma – real life experience

**DOI:** 10.1186/s12885-022-09490-8

**Published:** 2022-04-20

**Authors:** Barbara Ziółkowska, Bożena Cybulska-Stopa, Dimitrios Papantoniou, Rafał Suwiński

**Affiliations:** 1 Maria Sklodowska-Curie National Research Institute of Oncology, Gliwice Branch, Poland; 2 Maria Sklodowska-Curie National Research Institute of Oncology, Cracow Branch, Poland; 3 Department of Medical Sciences, Endocrine Oncology, Uppsala University, Uppsala, Sweden; 4 Department of Oncology, Ryhov County Hospital, Jönköping, Sweden

**Keywords:** Malignant pleural mesothelioma, Pemetrexed, Retreatment

## Abstract

**Background:**

Malignant pleural mesothelioma (MPM) is a rare, aggressive malignancy of the pleural cavity linked to asbestos exposure. The combination of pemetrexed and platinum is a standard first-line therapy for malignant pleural mesothelioma. Despite some progress, almost all MPM patients experience progression after first-line therapy. The second-line treatment is still being under discussion and there are very limited data available on the second-line and subsequent treatments.

**Methods:**

The retrospective analysis included 57 patients (16 females and 41 males) from two Polish oncological institutions treated for advanced mesothelioma between 2013 and 2019. We analysed the efficacy of first-line and second-line therapy: progression-free survival (PFS), overall survival (OS), overall response rate (ORR).

**Results:**

In the first-line treatment, 55 patients received pemetrexed-based chemotherapy (PBC) and two cisplatin in monotherapy. Patients’ characteristics at baseline: median age was 64.2 years, ECOG PS ≤ 1 (86.2%), epithelial histology (85.7%). Median PFS and OS were 7.6 months and 14 months, respectively. Patients with ECOG PS ≤ 1 vs > 1 had a longer median OS (14.8 months vs 9.7 months, *p* = 0.057). One-year OS and PFS were 60.9% and 32.0%, respectively. Disease control rate (DCR) was 82.5%. Response to first-line therapy: PFS ≥ 6 months and PFS ≥ 12 months had a significant impact on median OS. Twelve patients received second-line therapy (seven PBC and five other cytotoxic single agents: navelbine, gemcitabine, or adriamycin/vincristine/methotrexate triplet). Median PFS and OS were 3.7 months and 7.2 months, respectively. DCR was 83%. One-year OS and PFS were 37% and 16.7%, respectively.

In the group receiving PBC, OS was prolonged by 4.5 months compared to the non-PBC group (6.0 months vs 10.5 months, *p* = 0.47).

**Conclusion:**

Patients who benefited from first-line therapy and had prolonged PFS at first-line and achieve PFS longer than 6 months at first-line should be offered second-line treatment. Consideration of retreatment with the same cytotoxic agent could to be a viable option when no other treatment are available.

## Introduction

Malignant pleural mesothelioma (MPM) is a rare, aggressive malignancy of the pleural cavity linked to asbestos exposure. Despite recent treatment advances, prognosis remains poor with a median survival of approximately 1-year, and 5-year survival of around 10% [1–3]. The use of asbestos has been banned in many, but not all, countries. The incidence of MPM is increasing worldwide, mostly in Western Europe, China, Brazil, Russia, and India [3, 4]**,** and it is estimated that annually more than 25 000 people die from the disease [4]. The observed increase can be, thus, probably explained by a lag time of 30 – 45 years of the occurrence of MPM after exposure to asbestos [5].

Standard therapeutic approach for MPM includes surgery and chemotherapy. Adjuvant radiotherapy is not standard, and it can be considered only in the highly selected group of patients with good performance status and appropriate renal and pulmonary function [6, 7]. Unfortunately, at presentation, most patients are not eligible for surgical resection and palliative chemotherapy is the only possible strategy. Cisplatin plus pemetrexed doublet is the standard treatment as its activity had been proven in the EMPHACIS phase 3 trial [8]. Use of this combination significantly prolonged overall survival (OS) by 2.8 months and progression-free survival (PFS) by 1.8 months compared to cisplatin monotherapy. For elderly patients or those unfit to receive cisplatin, regimens containing carboplatin are an acceptable alternative [9]. Over a decade later, a phase 3 trial [10] showed the benefit of the addition of bevacizumab to pemetrexed/cisplatin, followed by maintenance treatment with bevacizumab, compared with chemotherapy alone. OS was prolonged by 2.7 months and this combination might be considered for patients eligible for triplet therapy. In October 2020, the U.S. Food and Drug Administration (FDA) approved the combination of nivolumab (anti–programmed death 1 agents, anti-PDL-1) and ipilimumab (anti–cytotoxic T-lymphocyte–associated antigen 4 monoclonal antibody, anti-CTLA-4) as first-line treatment for unresectable malignant pleural mesothelioma, based on results from the CheckMate743 phase III study. In the group assigned to anti-PDL1/anti-CTLA4 combination, the mOS was prolonged by 4 months compared to pemetrexed/platinum doublet [11, 12].

Despite some progress, almost all MPM patients experience progression after first-line therapy. There are very limited data available on the second-line and subsequent treatments. However, patients who benefited from first-line and have good Eastern Cooperative Oncology Group performance status (ECOG PS) are often offered further lines of treatment. A phase 3 trial showed the superiority of pemetrexed over best supportive care (BSC) in pemetrexed-naïve patients in terms of overall response rate (ORR) and PFS but not OS [13]. Data from case series suggest that in selected cases with good response to first-line pemetrexed-based chemotherapy (PBC), rechallenge with pemetrexed is an effective option [14–17].

Other chemotherapeutics such as vinorelbine [18] or gemcitabine [19] might be considered, but their efficacy is modest [20–22]. Recently, promising data from phase 2 studies have shown that immune-checkpoint inhibitors could be a viable option for MPM patients. National Comprehensive Cancer Network guidelines recommend the use of pembrolizumab or nivolumab with or without ipilimumab at or beyond the second-line of treatment [23, 24]. Numerous immunotherapy studies are ongoing and will hopefully lead to further changes in MPM treatment, as observed with other malignancies (www.clinicaltrials.gov).

The purpose of the present study is to evaluate the efficacy and tolerance of second-line treatment and retreatment with pemetrexed-based chemotherapy.

## Materials and methods

Data from all 164 MPM patients were collected at two Polish oncological institutions (*Maria Sklodowska-Curie National Research Institute of Oncology, Gliwice Branch and Cracow Branch*) between 2013 and 2019, and reviewed retrospectively. One hundred and six patients were referred for either second opinion or for palliative radiotherapy, and they were excluded from the analysis. Additionally, three patients received treatment in the adjuvant setting, and they were not included either. Fifty-seven MPM cases were identified with full clinical data and received first-line therapy. Patient characteristics are shown in Table 1.

**Table 1:** Patients characteristics

	No. of patients *N* = 57 (%)
**Sex**	
Female	16 (28)
Male	41 (72)
**Age (years)**	
Median, range	64.2 (33–83)
**ECOG performance status**	
0 9	(15.8)
1 41	(72)
2 7	(12.2)
**Histological subtype**	
Epithelial	42 (73.7)
Biphasic	5 (8.8)
Sarcomatoid	2 (3.5)
Not available	8 (14)
**Stage**	
III 45	(78.9)
IV	12 (21.1)
**Surgery:**	
yes	11 (20)
no	46 (80)
**First-line therapy**	57
Pemetrexed-based	55 (96.5)
With platinum	48 (87.3)
Without platinum	7 (12.7)
Not pemetrexed-based	2 (3.5)
With platinum	2 (100)
**Response to first-line therapy**	
CR/PR	20 (35.1)
SD	27 (47.4)
PD	9 (15.8)
vNot available	1 (1.7)
**PFS after first-line therapy**	
< 6 months	14 (24.6)
≥ 6 months	29 (50.9)
< 12 months	43 (75.4)
≥ 12 months	14 (24.6)
**Second-line therapy**	12
Pemetrexed-based	7 (58.3)
Non pemetrexed-based	5 (41.7)

*ECOG* Eastern Cooperative Oncology Group, *PFS* progression-free survival, *CR* complete response, *PR* partial response, *SD* stable disease, *PD* progression disease

For each patient the following data were collected: age, gender, histology, ECOG PS, treatment administered, first-line and second-line outcomes (PFS, OS, ORR), treatment-related toxicity. The planned number of cycles administered was six, regarding the tolerance, response to the therapy and patients’ decision. However, in peculiar situations, the therapy was continued beyond the sixth cycle (physicians individual decision). Best tumour response was evaluated according to the revised version RECIST 1.1 criteria.

## Statistical methods

PFS was calculated as the time from the start of systemic treatment until disease progression as confirmed by radiological evaluation, clinical examination, or until death from any cause. Patients without progression on the date of last follow-up were censored on that date. First-line PFS and second-line PFS were calculated separately. OS was defined as the time from the onset of treatment until death from any cause. Patients who were alive on the date of the last follow-up contact were censored. First-line OS and second-line OS were calculated separately. Treatment differences for PFS and OS were assessed using stratified log-rank test. Statistical analysis was performed with R version 3.5.3. PFS and OS were analysed using the Kaplan–Meier method. *P* values < 0.05 were considered statistically significant.

## Results

There were 16 females and 41 males with a median age of 64.2 years at diagnosis (range 33–83 years). The majority of patients had ECOG PS 0–1 (86.2%). The epithelial histological subtype was most common (72.4%). In our group, eleven patients (19%) underwent surgery: in three cases, extrapleural pneumonectomy (EPP) and in the other eight cases pleurectomy/decortication. All of them progressed thereafter and were referred for systemic therapy. None of them received adjuvant therapy. Fifteen patients (26%) who were not eligible for chemotherapy received radiotherapy in the palliative setting. None of the patients was a candidate for trimodality therapy.

### First line

**Fig. 1:**
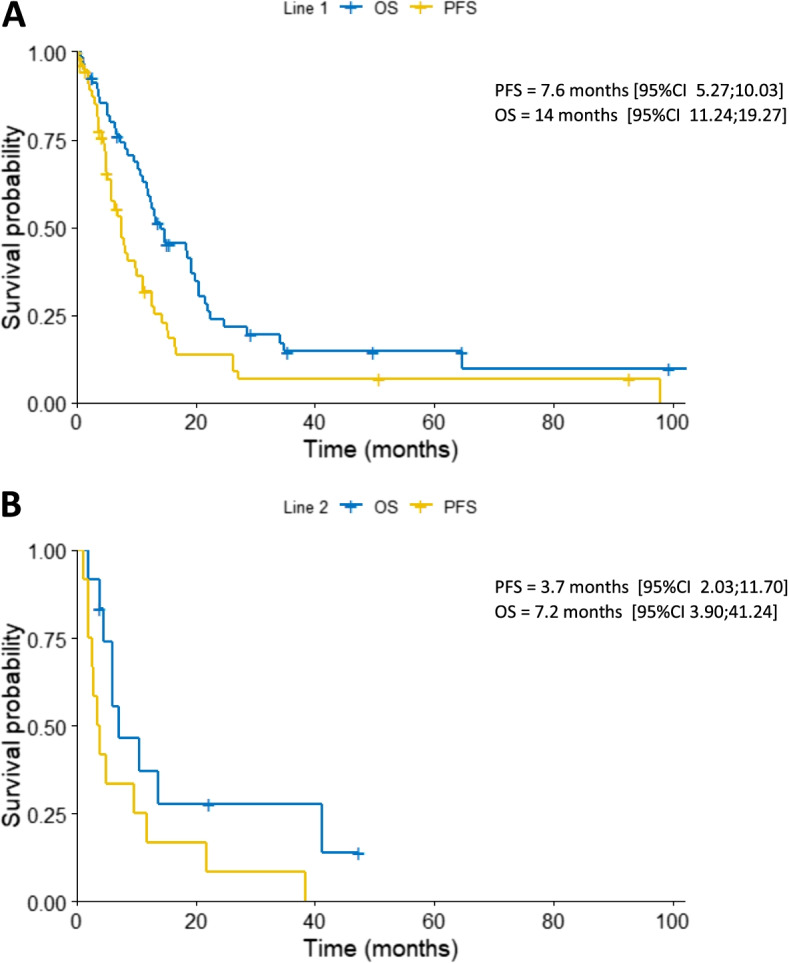
**A** Overall survival (OS) and progression-free survival of patients treated with first-line chemotherapy. **B** Overall survival (OS) and progression-free survival of patients treated with second-line chemotherapy

**Fig. 2:**
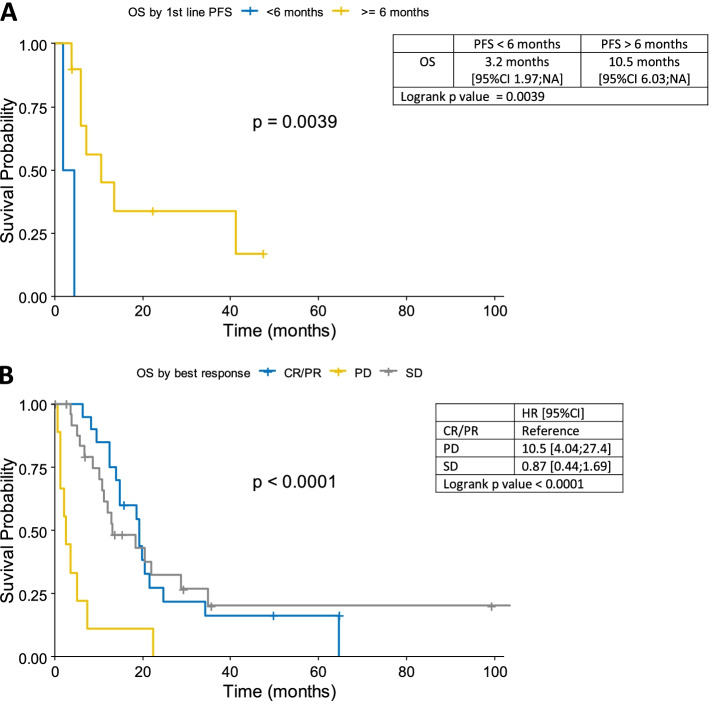
**A** Overall survival in patients stratified according to duration of progression-free survival (PFS) after first-line (FL) chemotherapy. Yellow line: PFS ≥ 6 months, blue line: PFS FL < 6 months. **B** Overall survival stratified according to response to first-line chemotherapy. Blue line: CR, complete response / PR, partial response; Grey line: SD, stable disease; yellow line: PD, progression disease

In the first-line setting, pemetrexed based chemotherapy (PBC) was administered to 55 patients: 48 patients received the combination of pemetrexed and platinum (45 with cisplatin, 3 with carboplatin), and seven patients pemetrexed in monotherapy. The remaining two patients were treated with single-agent cisplatin. A median number of 5 cycles was delivered (range 1–9). Thirty-one patients received the planned number of cycles. Six patients discontinued the treatment due to the deterioration of performance status, and 18 patients due to the progression of disease. The median PFS after first-line was 7.6 months (range 0–98 months), and the median OS was 14 months (range 0–140 months) (Fig. 1A). A longer median OS was observed in patients with ECOG PS ≤ 1 than > 1 (14.8 months vs 9.7 months, *p* = 0.057). One-year OS and PFS were 60.9% and 32.0%, respectively. Patients with longer responses at first-line had also a significantly longer median OS: i) when PFS ≥ 6 months vs < 6 months, median OS was 10.5 months vs 3.2 months (*p* = 0.0039) (Fig. 2A), ii) when PFS ≥ 12 months vs < 12 months, median OS was 41.2 vs 6.0 months (*p* = 0.039). Disease control rate (DCR) defined as the sum of CR, PR, and SD, was 82.5%. Twenty patients (35.1%) responded to first-line chemotherapy (CR in 1 and PR in 19 cases), whereas 27 patients (47.4%) achieved SD (Fig. 2B). We did not observe any difference in terms of histological subtype (*p* = 0.22) probably due to a small group of patients.

The grade 1 or 2 toxicities were primarily hematologic and were manageable. They included anaemia (25%), neutropenia (13%), fatigue (12%), loss of appetite (9%), and deterioration of renal function defined as a decrease of the *estimated glomerular filtration rate under 60 ml/min/1.73 m2* (9%). Grade 3 anaemia occurred in 1 patient only. We did not report any death related to the treatment.

### Second line

Among 50 patients who progressed after first-line chemotherapy, 12 (24%) were eligible for second-line treatment regarding their performance status (Table 2). All of them received cisplatin/pemetrexed doublet in the first-line. Seven patients were retreated with PBC (1 with pemetrexed in monotherapy and 6 with a pemetrexed/cisplatin combination). A median number of 5 cycles was given (range 2–6). In 5 cases a non–pemetrexed therapy was used, which consisted of single-agent gemcitabine in 2 cases, single-agent vinorelbine in 2 cases, and a triplet of adriamycin/vincristine/methotrexate in 1 case (Table 3). Seven patients received treatment as planned. Two patients discontinued the therapy due to the unacceptable toxicity, and three patients due to the disease progression. The median PFS after second-line was 3.7 months (range 1.2–38.4 months), and the median OS was 7.2 months (range 2.0–47.3 months) (Fig. 1B). In the group receiving PBC, OS was prolonged by 4.5 months compared to the non-PBC group (6.0 months vs 10.5 months, *p* = 0.47). The difference was more apparent when doublet pemetrexed/platinum was compared with single-agent therapy: median PFS was 7.3 months vs 3.0 months (*p* = 0.53), and median OS was 12.1 months vs 4.5 months (*p* = 0.17). Patients who received the second-line therapy regardless of the regimen administered had a significantly prolonged median OS compared the those who did not receive it (12.5 months vs 21.9 months, *p* = 0.031).

**Table 2:** Patients characteristics in the second-line treatment

	**All patients** ***n* = 12**	**Pemetrexed-based therapy** ***n* = 7**	**Pemetrexed-based therapy** ***n* = 5**
**Sex**			
Female	2	1	1
Male	10	6	6
**Age (years)**			
Median, range	61.7 (50–79)	65.3 (50–79)	56.5 (42–68)
**ECOG**			
0	2	1	1
1	10	6	4
**Histological subtype**			
Epithelial	10	5	5
Sarcomatoid	1	1	0
Biphasic	1	0	1
**Surgery**			
Yes	3	2	1
No	9	5	4
**First-line therapy**			
Pemetrexed-based	12	7	6
Not pemetrexed-based	0	0	0
**Best response to first-line**			
CR/PR	5	4	1
SD	6	3	3
PD	1	0	1
**PFS after first-line therapy**			
< 6 months	1	0	1
6– 12 months	7	4	3
> 12 months	4	3	1

*ECOG* Eastern Cooperative Oncology Group, *PFS* progression-free survival, *CR* complete response, *PR* partial response, *SD* stable disease, *PD* progression disease

**Table 3:** Detailed second-line therapy and PFS from first- and second-line therapy in patients who received second-line

Patient No	PFS from first-line (months) Cisplatin/Pemetrexed	Second-line therapy	PFS from second-line (months)
1	7.1	Gemcitabine	1.2
2	10.1	Cisplatin/Pemetrexed	5.1
3	10.9	Cisplatin/Pemetrexed	2.9
4	11.1	Cisplatin/Pemetrexed	9.8
5	13	Vinorelbine	22.1
6	16.8	Cisplatin/Pemetrexed	11.9
7	26.3	Cisplatin/Pemetrexed	2.1
8	44	Cisplatin/Pemetrexed	39.0
9	5.3	Adriamycin/Vincristine/Methotrexate	2.7
10	7	Gemcitabine	3.5
11	8.7	Cisplatin + Pemetrexed	2.1
12	8.1	Vinorelbine	4

All patients were evaluated for best tumour response. Disease control was confirmed in 83% case. PR was achieved in 1 case (in PBC group), nine patients (75%) achieved SD (5 in PBC group and 4 in the non-PBC group), and the remaining two progressed. Three patients received therapy beyond the second-line. Two of them were given third-line (in 1 case single-agent gemcitabine, and the other one was retreated again with pemetrexed/cisplatin). One patient was given six lines of treatment: vinorelbine in the third-line, gemcitabine in the fourth-line, pemetrexed/carboplatin in the fifth-line and then rechallenge with pemetrexed/carboplatin in the sixth-line. At a median follow up of 8 months (0–139.4 months) 49 patients had died, and seven are still alive without any evidence of disease progression. In one case data are missing. One-year OS and PFS were 37% and 16.7%, respectively.

The grade 1 or 2 toxicity in the second-line therapy included anaemia (58%), neutropenia (25%), fatigue (41%), polyneuropathy (24%), and deterioration of renal function defined as a decrease of the *estimated glomerular filtration rate under 60 ml/min/1.73 m2* (25%). Treatment-related adverse events that led to the discontinuation of therapy occurred in 2 patients (anaemia grade 3 and polyneuropathy grade 2). Neither grade 4 toxicity nor treatment-related deaths were reported.

## Discussion

Pleural malignant mesothelioma is a very aggressive malignancy with poor prognosis even after radical therapy. Due to an advanced stage at presentation in the majority of MPM patients, chemotherapy is the only strategy to be offered. In this report, we presented the results of the systemic treatment for MPM from two Polish institutions. The pemetrexed-based therapy was administered in the first-line setting to 57 patients. First-line median PFS and OS were 7.6 months and 14.0 months, respectively, with a DCR of 82.5%. After one year, 32% of all cases were progression-free and 61% still alive. The therapy was relatively well tolerated, and dose reduction was required only in seven cases (12%). These results are consistent with papers from the registration trial for pemetrexed in MPM conducted by Vogelgang et al. [8].

There are still very limited data on the second and subsequent lines of chemotherapy for MPM, but younger patients with good ECOG PS who benefited from first-line might be considered as candidates for second-line therapy when progression occurs. A phase 3 trial [13] comparing pemetrexed vs best supportive care in second-line in pemetrexed-naïve MPM patients demonstrated improvement in DCR and PFS, but not OS. It was interpreted as a result of lack of balance between groups and the fact that in the best supportive care arm the radiotherapy was allowed. A post hoc multiple regression analysis of the post-study chemotherapy adjusted for group imbalances [25] showed a significantly longer OS compared with best supportive care. Similarly, we also observed markedly prolonged median OS when second-line therapy was administered.

In our study group, 12 (24%) out of 50 patients who progressed after first-line were eligible for second-line. The type of therapy was the physician’s choice according to the local preferences. In 58% of cases retreatment with pemetrexed was given, and the remaining 42% of patients received other cytotoxic agents. In the radiological assessment, 10 out of 12 patients achieved clinical benefit (DCR = 83%). Overall, second-line median PFS and median OS were 3.7 months and 7.2 months, respectively. Subgroup analysis demonstrated median OS prolonged by 4.5 months in the group receiving PBC compared with other cytotoxic agents (6.0 months vs 10.5 months, *p* = 0.47). We observed a trend in favour of pemetrexed, especially when doublet containing platinum was given compared with single agents: median PFS 7.3 months vs 3.0 months (*p* = 0.53) and median OS 12.1 vs 4.5 months (*p* = 0.17) but the results were not statistically significant.

There are few reports on the pemetrexed rechallenge after progression to first-line. Razak [26] and Hayashi [27] presented small case series of 4 patients each retreated with PBC. They achieved outstandingly prolonged PFS ranging from 23–73 months [26], and 6.4–11.4 months [27]. The biggest cohorts were presented by a French group [28] and in 2 retrospective Italian multicentre studies. Their populations had different baseline characteristics, choice of second-line, or medications used in first-line. Nevertheless, the results are consistent with ours. Groups presented by Zucali [16] and Ceresoli [15] were partially overlaying. Zucali analysed the second-line therapy in MPM patients where almost 34% of patients were pemetrexed-naïve after first-line and 23% did not receive PBC in second-line. Patients retreated with pemetrexed ucontaining regimens achieved significantly longer PFS and OS compared with non-PBC therapy in second: PFS 6.2 months vs 2.8 months (*p* = 0.006) and OS 10.6 months vs 7.0 months (*p* = 0.028). Ceresoli et al. [15] enrolled 32 patients who achieved disease control (PR/SD) lasting for at least 3 months after first-line PBC. Eighteen of them were retreated with PBC in second-line, and they achieved a median PFS and median OS 3.8 months and 10.5 months, respectively. In another Italian cohort [14] 30 patients pretreated with PBC received pemetrexed in the second-line setting (monotherapy/combination with platinum). Median PFS and OS were 5.1 months and 13.6 months, respectively, while DCR was 66%.

In accordance to the data from several other studies [14–17, 25–27] (www.clinicaltrials.gov), we also observe that there might be a correlation between the response to first-line and the benefit from second-line. Patients who had a longer response to the first-line (PFS ≥ 6 months vs PFS < 6 months) had a significantly prolonged median OS (*p* = 0.0039). This difference was even longer when PFS ≥ 12 months (*p* = 0.039).

We are aware that our study has several limitations typical of retrospective research. Also, the number of patients is small and restricts the power of comparisons between treatment groups. However, this is one of the only few studies that examine the outcome of second-line treatment in this rare disease.

## Conclusion

Our data support previous results for second-line therapy in MPM. We conclude that patients who benefit from first-line therapy and achieve PFS longer than 6 months at first-line should be offered second-line treatment. Retreatment with the same cytotoxic agent seems to be a viable option, especially regarding pemetrexed/platinum combination, particularly whenever no alternative therapy is available. Those who do not achieve PFS longer than six months with first-line therapy but are in a good performance status should also be offered the second-line therapy using other cytotoxic agents.

## Data Availability

The data are available from the corresponding author on reasonable request.
